# Diagnosis a posteriori? Assessing gestational diabetes screening and management in Morocco

**DOI:** 10.3402/gha.v9.32511

**Published:** 2016-11-17

**Authors:** Bettina Utz, Bouchra Assarag, Amina Essolbi, Amina Barkat, Yassir Ait Benkaddour, Vincent De Brouwere

**Affiliations:** 1 Public Health Department, Institute of Tropical Medicine, Antwerp, Belgium; 2 National School of Public Health, Rabat, Morocco; 3 Faculty of Medicine and Pharmacy, Mohammed V University, Rabat, Morocco; 4 Faculty of Medicine, Cadi Ayyad University, Marrakesh, Morocco

**Keywords:** maternal health, Morocco, diabetes, pregnancy, screening, management

## Abstract

**Background:**

In Morocco, gestational diabetes affects 1 in 10 pregnant women, but knowledge about screening and management practices outside university settings is limited.

**Objective:**

To provide a comprehensive picture about the current situation of screening and management of gestational diabetes at different levels of care and to highlight existing challenges.

**Design:**

We conducted a descriptive mixed methods study in the districts of Al Haouz and Marrakech by using both quantitative and qualitative methods, including document reviews of 369 antenatal cards and 299 hospital files, health facility inventories related to resource availability, 20 key informant interviews as well as focus group discussions with 32 pregnant women and exit interviews with 122 antenatal care (ANC) clients. Quantitative data were descriptively analyzed using STATA Version 13, whereas qualitative data were thematically analyzed using NVIVO Version 10.

**Results:**

The findings revealed that sensitization of women about gestational diabetes is low, and only 34.4% have ever heard about it before attending ANC. Fasting blood sugar is used for screening, and women are sent to external laboratories for testing. A fasting blood sugar of 0.92 g/l and above was documented in 12.3% of all antenatal cards examined. Women diagnosed with gestational diabetes are usually referred to a specialist despite general practitioners at health center level being responsible for the management of non-pregnant diabetic patients.

**Conclusions:**

Decentralization of screening for gestational diabetes and initial management of uncomplicated cases at the primary level of care could ease access to care and reduce the number of mothers who are diagnosed after a complication occurred.

## Introduction

Diabetes, derived from the Greek word *diabainein*, to pass through, is literally traversing the globe, affecting not only people in high-income countries but also progressively in low- and middle-income countries. Between 1990 and 2013, the age-standardized rate increased by 44.8%, the highest change observed among 301 health problems analyzed (1). In 2013, diabetes accounted for a total of 29.5 million years lived with disability (YLD) and ranked as seventh top 10 causes of YLD in developing countries (1). Simultaneously, we observe an increase in pregnant women who develop gestational diabetes mellitus (GDM), with worldwide already 15 of 100 pregnant women being affected (2). Their risk to experience a complication during pregnancy or at birth such as preeclampsia or eclampsia, postpartum bleeding, and prolonged labor or to develop a long-term chronic condition is higher compared with healthy pregnant women. Moreover, their newborns are more prone to die around the time of birth, to be born prematurely or with malformations, or to be large for gestational age, with the additional risk of the delivery being complicated by a shoulder dystocia that can result in a plexus injury or a fracture (3, 4). Newborns might suffer from asphyxia, respiratory distress, or hypoglycemia (5) and also may develop metabolic abnormalities later in life (6). By establishing a timely and adequate management of gestational diabetes, the risk for aforementioned complications could be reduced. Furthermore, screening in pregnancy serves the additional purpose as a first step in the prevention of diabetes later in life by establishing a closer surveillance of individuals at risk (7).

In Morocco, reported GDM prevalence ranges between 8.2 and 10% (8, 9), but current population-based national figures are unknown. Taking into account an increased risk for obese women to develop GDM (10), the actual prevalence of GDM might be even higher in Morocco, where already 55% of women aged 25–44 years are overweight or obese (11). Despite a substantial number of affected women, there is to date no research being conducted that examines the actual situation of screening and management of GDM in Morocco outside larger university settings. National best practice guidelines for gestational diabetes exist and although these guidelines include information on screening, messages in the different protocols are not uniform and there is very limited information about their practical application in health facilities. We conducted this situational analysis in the districts of Marrakech and Al Haouz to assess the current situation of gestational diabetes screening and management and its related challenges for providers and pregnant women. It serves as a basis for the development of a national GDM strategy that is adapted to the local context, enabling universal access to detection and care. This is in line with the aspiration of the Global Alliance for Chronic Diseases to ‘tackle the burden of chronic non-communicable diseases in low- and middle-income countries by systematically building the evidence base for sound policymaking’ (12).

## Methods

We did a descriptive mixed methods study using a concurrent triangulation design (13) in the districts of Al Haouz and Marrakech between June and December 2015. Both districts together have a population of 1,903,000, with the district of Marrakech being essentially urban and the neighboring district of Al Haouz being predominantly rural and with poor accessibility to health care facilities because of its mountainous landscape.

The study was conducted at all three levels of care: public health centers and private clinics (primary level), the regional hospital (secondary level), and the university hospital (tertiary level). We randomly selected 15 public health centers stratified by type (with/without a maternity ward), location (urban/rural), and number of antenatal care (ANC) visits per month (above/below 30). We also included three randomly selected private clinics in Marrakech (*n*=2) and Al Haouz (*n*=1) as well as the regional and university hospitals (*n*=2). The data collection tools consisted of standardized forms for general and individualized data, topic guides for qualitative interviews, and an exit survey questionnaire adapted from existing client interview guides (14). These tools had been developed in close collaboration with members of the Moroccan GDM research group composed of researchers, clinicians, and representatives of the Ministry of Health and were piloted in health facilities of different levels in Rabat. In each primary health care facility and in the private clinics, information on staff, number of monthly ANC visits, availability of antidiabetic drugs and consumables, and availability of protocols was collected. From a random sample of ANC files of women attending ANC in the survey period, we collected information on documented risk factors for GDM and recommended and performed tests related to GDM screening and on the management of positively tested women. Based on our assumption that at least half of all pregnant women attending ANC are tested for glycemia in pregnancy, we calculated a required sample size of 300 women (at a confidence level of 95%) by using the two-sided *t*-test for proportions (PASS software Version 12). In each public and private health care center included, we assessed between 19 and 24 cards, with a median of 20 cards per facility resulting in data collected from a total sample of 369 patients. Furthermore, 122 women attending ANC services in the surveyed facilities (between 5 and 22 women per facility) gave their consent to participate in a short, structured exit interview containing 18 closed questions to assess information asked/received during their ANC visit with regard to GDM.

In the regional and the university referral hospitals, we reviewed all the maternity hospital files completed during the period between June and December 2015 and extracted 299 files of women with a GDM/diabetes or who presented with complications potentially associated with GDM such as the birth of a macrosomic baby (equal or more than 4,000 g), a premature delivery, referral of the newborn to intensive care, a delivery complicated by shoulder dystocia, preeclampsia, and primary caesarean sections to assess how many of these women were affected by GDM/diabetes and if peri- and postpartum glycemia testing occurred.

To learn about current policies, practices, and challenges related to GDM screening and management, we purposively selected 20 key informants based on their involvement in maternal health/diabetes programs on national, regional, and local levels, including representatives of the Ministry of Health at the central (*n*=3) and regional (*n*=7) levels, informants actively involved in screening and care provision of pregnant women in the public (*n*=6) and private health care sector (*n*=2) as well as representatives of associations involved in diabetes sensitization (*n*=2). Four short focus group discussions with a total of 27 pregnant women attending ‘women's classes’, information sessions for pregnant women, and a discussion with 5 women affected by gestational diabetes to assess their knowledge about GDM and experiences complemented our analysis.

Quantitative data were collected on standardized data collection forms, entered into a preformatted Excel spreadsheet and converted for further data processing into STATA Version 13. After data cleaning, we analyzed the data by using means and standard deviations for normally distributed continuous variables and by summarizing categorical variables as counts and percentages. Bivariate analyses were performed, and relationships between categorical variables were examined using the Pearson's chi-square test. Qualitative data were translated, transcribed and thematically analyzed using NVIVO 10.

Ethical approval for the study was granted by the Ethics Committee for biomedical research at the University Mohammed V, Rabat, Morocco (Dossier 61/15); the Institutional Review Board at the Institute of Tropical Medicine; and the Ethics Committee of the University Hospital Antwerp, Belgium (1007/15 and B 300201525040).

## Results

Both qualitative and quantitative results have been organized according to four thematic areas: sensitization, screening at ANC, management of GDM and GDM-related birth complications, and postpartum diagnosis to address the continuum of care regarding gestational diabetes in Morocco.

### Sensitization

**Fig. 1:**
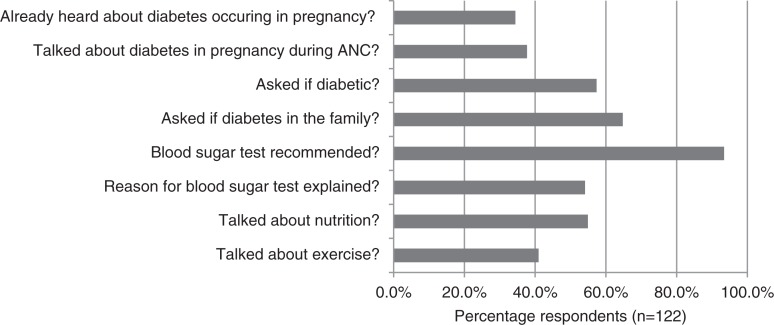
Information received during exit interviews with 122 women attending antenatal care (ANC).

The results of 122 ANC exit interviews with pregnant women revealed that 34.4% (*n*=42) of them have never heard about gestational diabetes before, whereas 37.7% (*n*=46) of women said they heard about gestational diabetes at the ANC clinic. Overall, 93.4% of ANC clients (*n*=114) stated they had been asked to have a fasting blood sugar test done, but only 54.1% (*n*=66) received an explanation about the reasons for testing (Fig. 1).

Some of the above-mentioned findings were also reflected in the focus groups with pregnant women who criticized that despite being told to have their blood sugar measured, they were not informed about the reasons. ‘For example this blood sugar test: no-one has explained that to us like today; we were asked to do the analysis, we do it, she tells us “we will see the results”’ (Pregnant woman, FGD). Women would like to receive more information about gestational diabetes and want to be able to ask questions. ‘We would like to be informed before having the problem and come and ask questions’ (Pregnant woman, FGD). But sensitization about gestational diabetes is often not done, and some women do not even know that they are routinely tested for diabetes in pregnancy. ‘We would very much like that someone explains us and also tests if we have diabetes or not. But no one has ever talked to us about that’ (Pregnant woman, FGD).

### Screening for GDM

Protocols with information on gestational diabetes screening were available in 14 (77.8%) of 18 primary public and private facilities assessed. In accordance with the Moroccan ANC guidelines that recommend a fasting blood sugar both in the first and in the second trimester (15), a fasting blood sugar was usually prescribed at the first contact with the pregnant women during ANC, corresponding to information provided by a key informant ‘Fasting glucose is systematically requested of all pregnant women attending a health care facility during their first visit in the first trimester’ (Regional health program manager).

**Fig. 2:**
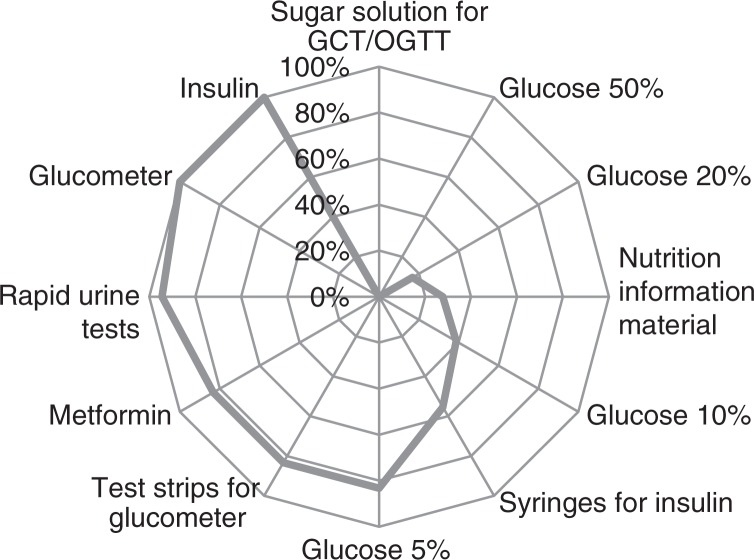
Availability of consumables and drugs with regards to gestational diabetes mellitus (GDM) screening and management in 18 public and private health centers. GCT, glucose challenge test; OGTT, oral glucose tolerance test.

Although glucometers were available at all facilities and respective test strips at 83% (*n*=15) of the assessed 18 public and private health centers (Fig. 2), women were generally sent to a laboratory to have a fasting glucose done as part of a wider range of tests including blood grouping, hemoglobin, testing for syphilis, toxoplasmosis, and rubella. However, women first had to make an appointment at the laboratory. The day of the appointment could take a couple of weeks as indicated by one medical doctor working at a health center: ‘The laboratory is at the regional hospital […]. They have a particular organization because they work by appointment. That's a problem… They have a quota of women who benefit from these tests. But they cannot meet all the needs which results in a delay that affects the tests and the screening’ (Medical doctor).

Even though lab tests in the public sector are free of charge, problems of access urge women to divert to the private sector. ‘It's free of charge on the papers, but in reality it is not free of charge because there are obstacles. For the ministry, the delivery is free of charge, they have introduced that the lab analyses are free of charge, but sometimes patients encounter difficulties, they are asked to do a lab test and they are obliged to do it in the private sector’ (Obstetrician-gynecologist).

**Fig. 3:**
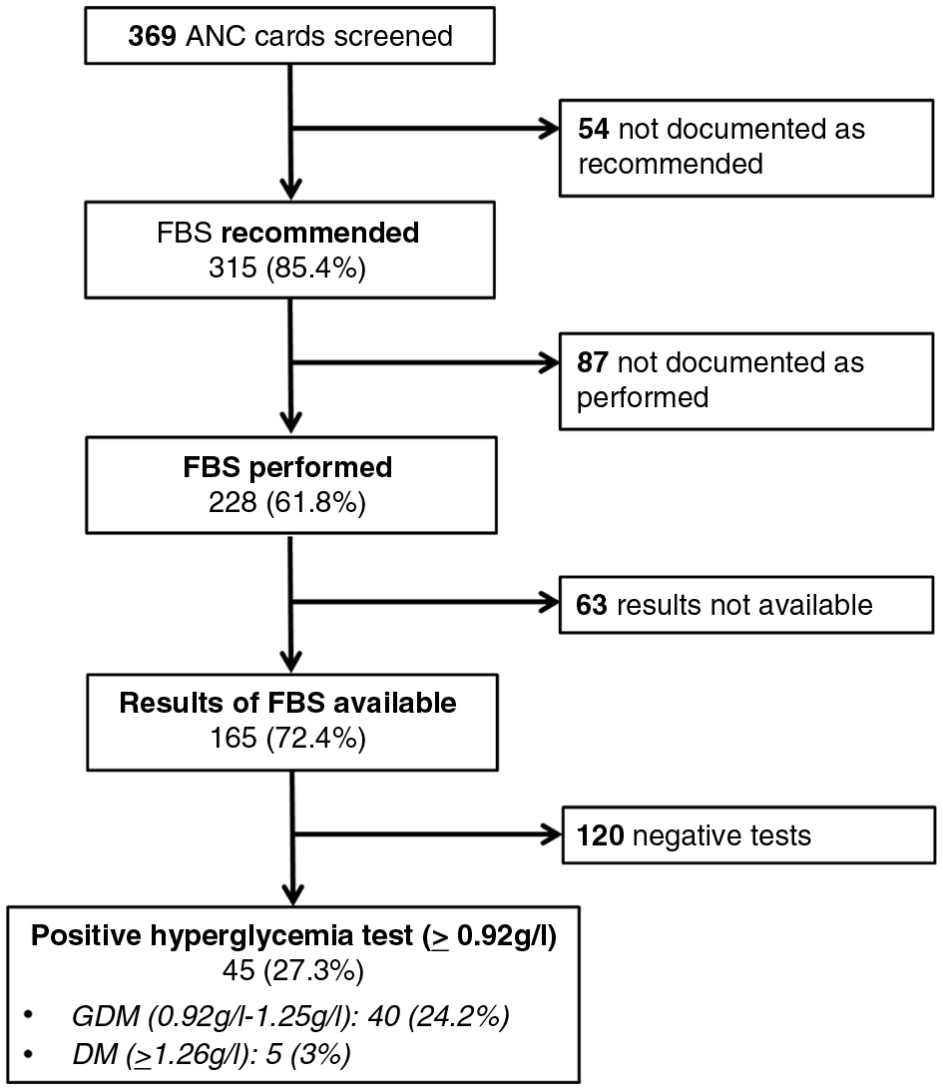
Documentation of diabetes screening in 369 antenatal care (ANC) cards. FBS, fasting blood sugar.

The analysis of ANC cards of 369 clients attending 15 public and 3 private health care centers (see Table 1 for client characteristics) revealed that a fasting blood sugar was recommended in 85.4% of all ANC cards (*n*=315) and documented as performed in 61.8% of cards (*n*=228). Test results were available in 72.4% (*n*=165) of these files. A fasting glucose of between 0.92 and 1.25 g/l, the diagnostic criterion for a gestational diabetes, was documented in 24.2% (*n*=40) of ANC cards and a fasting glucose of 1.26 g/l and above, the diagnostic criterion for a manifest diabetes mellitus, was reported in 3% (*n*=5) of files (Fig. 3). Although a higher proportion of tests were performed by educated women and women from urban settings, no significant differences were found between residence (urban/rural), educational level, and parity regarding a hyperglycemia level indicative of a gestational diabetes (Table 2).

**Table 1:** Characteristics of ANC attendants

Characteristics of ANC attendants		Total *N* (%)
Age (*n*=368) (years)	<20	37 (10.1)
	20–24	90 (24.5)
	25–29	94 (25.5)
	30–34	86 (23.4)
	35 +	61 (16.6)
Residence (*n*=369)	Urban	168 (45.5)
	Rural	201 (54.5)
Education (*n*=365)	None	113 (31.0)
	Primary	143 (39.2)
	Secondary	86 (23.6)
	Tertiary	23 (6.3)
Parity (*n*=356)	0	145 (40.7)
	1	101 (28.4)
	2–4	103 (28.9)
	5+	7 (2.0)
No. of ANC visits (*n*=369)	1	108 (29.3)
	2	127 (34.4)
	3	79 (21.4)
	4+	55 (14.9)
Mean GA at ANC visit (weeks)	1 (*n*=363)	17.5 (SD 7.1)
	2 (*n*=258)	25.3 (SD 6.4)
	3 (*n*=134)	31.6 (SD 5.1)

ANC, antenatal care; GA, gestational age.

**Table 2:** FBS performance and results in relation to residence, education, and parity of ANC attendants

Characteristics	FBS performed *N* (%)	*p*	Test positive for hyperglycemia (GDM) *N* (%)	*p*
Residence	(*n*=228)	<0.005	(*n*=40)	0.290
Urban	124 (54.4)		24 (60.0)	
Rural	104 (45.6)		16 (40.0)	
Education	(*n*=225)	<0.005	(*n*=40)	0.592
No	51 (22.7)		13 (32.5)	
Primary	94 (41.8)		17 (42.5)	
Secondary and more	80 (35.6)		10 (25.0)	
Parity	(*n*=222)	0.983	(*n*=39)	0.125
0	91 (41.0)		18 (46.2)	
1 to 2	104 (46.9)		14 (35.9)	
3 and more	27 (12.2)		7 (18.0)	

FBS, fasting blood sugar; ANC, antenatal care.

The diagnosis of a gestational or preexisting diabetes had been explicitly mentioned in only a quarter (26.7%; *n*=12) of all files, with a recorded fasting blood sugar of 0.92 g/l or above. Although glycemia results in the ANC cards were documented as fasting values, a challenge to the validity of these results may be the long distances women have to travel to get their blood tested, with the consequence that they might not be in a fasting state anymore upon arrival. A travel duration of 5 h or more to reach a laboratory was reported by a key informant. ‘We have a problem in the regions that are very far: the women leave their homes around six o'clock in the morning and arrive only around 11 o'clock, 11.30 or 12’. (Laboratory technician). In contrast, time to reach a health center was short and of 364 women in our sample, 59.3% (*n*=216) took<30 min to reach their health center, 26.6% (*n*=97) traveled between 30 and 60 min, and only 14% (*n*=51) took longer than 1 h.

### Management

Overall, in 18 patients with a documented GDM, further management procedures were highlighted and included diet or glucose control (22.2%; *n*=4), medical treatment with insulin (33.3%; *n*=6) or metformin (5.6%; *n*=1), or referral to an endocrinologist (38.9%; *n*=7). In Morocco, women with gestational diabetes are categorized as high-risk patients and have to be referred to a specialist, either to an endocrinologist or to an obstetrician-gynecologist according to key informants.

First, the endocrinologist is mandatory […], generally through a consultation in the private sector either in Marrakech or elsewhere. It is him who defines first the protocol, the number of units to inject, how often, how frequent and all that is needed. So first a consultation with the specialist and afterwards the follow-up at the level of the health centers. (Regional health program manager)

In 86.7% (*n*=13) of the 15 public primary health care facilities, providers indicated that pregnant women with a detected hyperglycemia are usually first presented to the general medical doctor based at the facility who decides on the further management and the referral needs. In 13.3% (*n*=2) of the facilities, providers reported to refer directly to a gynecologist or an endocrinologist as indicated by a midwife ‘When we detect a woman with gestational diabetes, we send her to the university hospital to do her follow-up […] She takes an appointment with a gynecologist who sees her and provides her with a treatment’ (Midwife).

However, particularly in the larger referral hospitals, adequate care of such patients cannot necessarily be guaranteed due to a high workload of the specialists.

You know, when you are a gynecologist at the hospital, you almost only treat emergencies. We do not provide a good pregnancy follow-up, because we do not have the files with all the information, there is nothing, frankly speaking. Often we receive them in a catastrophic condition while on duty. But even when we have the opportunity to detect them earlier, we don't have the time to establish a good ‘rapport’ with the patient to be able to educate, to explain the situation, to show the long-term consequences of gestational diabetes, to tell them that they need a life-long follow-up. (Obstetrician-gynecologist)

Furthermore, access to specialist services can present a major obstacle as getting an appointment takes time.

You have to calculate a minimum of 3 weeks, 1 month…[…] I had problems with women because we asked them to do the follow-up with the endocrinologist who is in Marrakech and they told us that it is not possible for them, the distance and everything. (Medical doctor)

But even if referral to a specialist poses no problem, there seem to be communication issues between the different levels of care that may affect adequate follow-up.

There is not much communication between the first level where they make the diagnosis and the second level where they treat. And even when they see the woman [at the first level] they send her straight to the secondary level of care. There is no feedback; there is no return of information from the specialist to the medical doctor to know what he should to do next. (National health program manager)

Apart from communication issues between the different levels, we also heard about inadequate client–provider interaction. Some women who were affected by GDM expressed their disappointment with the way some of the health care providers communicated with them.

Yes, I take for example 40 in the morning and 30 at lunch and at dinner. This was not sufficient and I went to the health center. They asked me ‘What do you want us to do? […]We can't do anything else’. (Woman affected by GDM, FGD)

Helplessness or indifference in providing support coupled with a lack of sympathy on provider side might threaten the relationship and the trust in health care providers.

When I came to see the doctor and I told her I can't afford to buy things she said: ‘I don't understand that you cannot afford to buy; it's not of my concern’. (Woman affected by GDM, FGD)

### Diagnosis a posteriori

Challenges related to timely diagnosis and adequate follow-up of women affected by GDM can result in women finally presenting with a complication at birth as described by a midwife.

During my work as midwife at the Maternity ward, I delivered women with gestational diabetes. […] Once I received a woman. She was fully dilated, head at the perineum. I suspected a diabetes because she had an excessive uterine height. […] I suspected she had a macrosomic baby. It was a difficult delivery […]. The weight of the baby was five, nearly five kilos. […] She was not followed up. These are women from rural settings, sometimes they just come for the delivery […]. (Midwife)

**Fig. 4:**
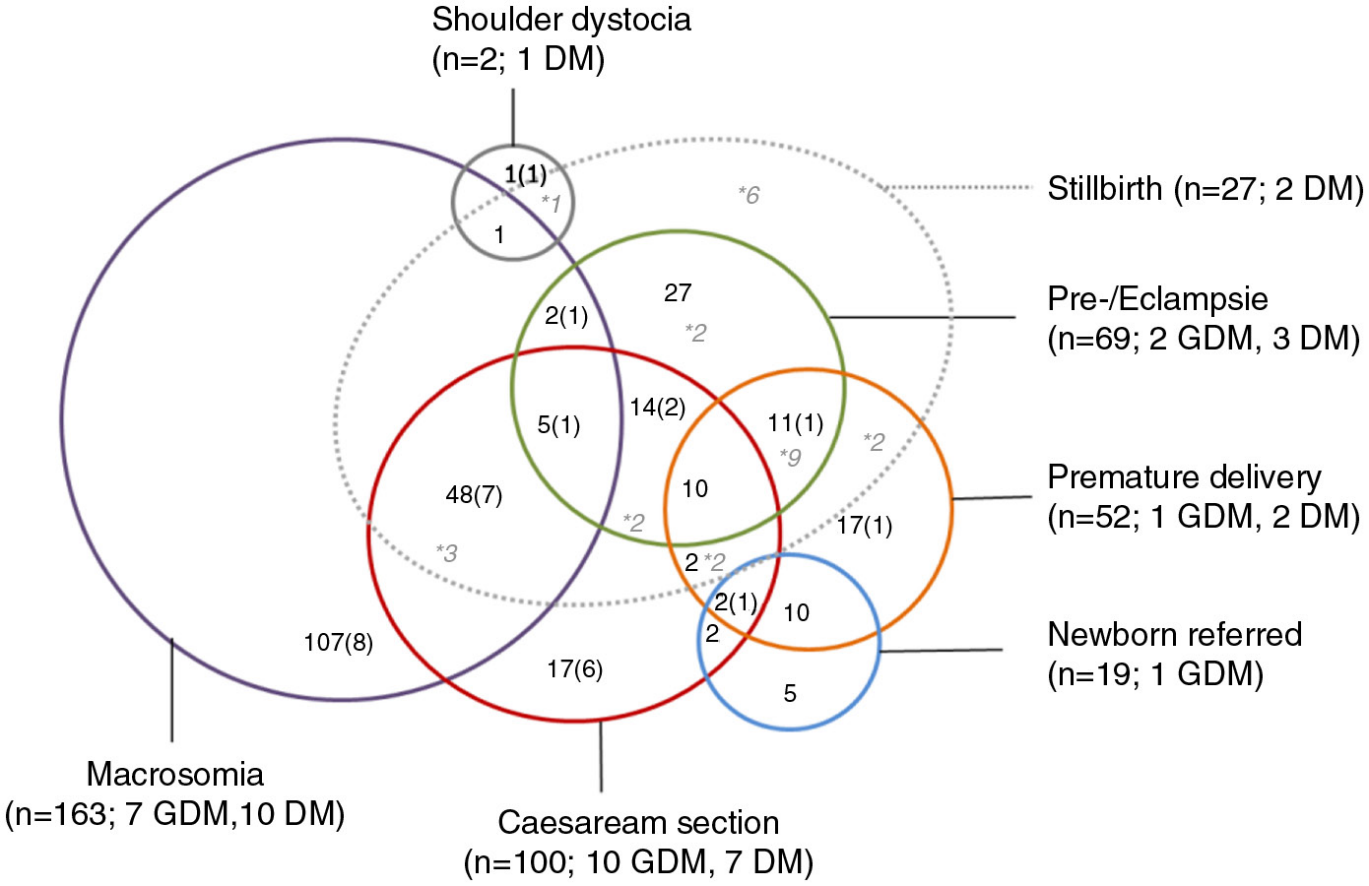
Complications potentially associated with gestational diabetes mellitus (GDM) (regional hospital, *n*=147; university hospital, *n*=152); in brackets documented cases of gestational diabetes mellitus/diabetes mellitus (GDM/DM); *number of stillbirths; [size of circles not corresponding to case numbers].

The study of 299 files of women with GDM/diabetes giving birth in the two urban referral hospitals or who experienced a complication potentially associated with GDM between June and December 2015 revealed that in 13% of the files (*n*=39), a manifest diabetes (7%; *n*=21) or a GDM (6%; *n*=18) was documented. Of these women, 25.6% (*n*=10) had a normal delivery, whereas 74.4% (*n*=29) experienced a complication known to be associated with a diabetic condition. In our sample, the majority of files were of macrosomic births (54.5%; *n*=163), and 48% of these deliveries were conducted by caesarean section. An overview of the different complications is provided in Fig. 4. A maternal blood sugar taken postpartum was recorded in 2% of the files (*n*=6), with most measurements of women who gave birth to a macrosomic baby (*n*=4; 66.7%). The above-mentioned findings indicate that postpartum maternal glycemia control for diagnostic reasons seem to be rarely performed in complications associated with GDM. However, a posteriori diagnosis of GDM, particularly in case of macrosomia, was indicated by key informants.

But generally, I know there are many women who just arrive for delivery and this is when diabetes is discovered. And sometimes even the woman is not aware. And when they see the big belly, they assume that this might be a diabetes. Only then, at that moment they test the blood sugar […]. (National health program manager)

## Discussion

The findings of our situational analysis regarding gestational diabetes screening and management in Morocco revealed positive aspects of current practices, but equally some existing challenges. Being part of a routine blood examination in pregnancy, a glycemia test is performed free of charge in the public laboratories. According to our findings, health care workers recommended the test for the majority of pregnant women. However, it is important to mention that a quarter of ANC cards containing a glycemia result showed values indicative of gestational or manifest diabetes. The reliability of the performed tests cannot be judged retrospectively, and we cannot rule out selection bias of women sent for testing. Verification of the high percentage of documented hyperglycemia would therefore require a prevalence study at the primary level of care.

More urban and educated women went for testing and although this could be partially due to residence and education acting as potential confounders, it may also indicate that financial and geographical access to blood tests play a role in Morocco, where faster appointments are available in the private for profit laboratories that are predominantly located in urban areas. According to the latest International Federation of Gynecology and Obstetrics recommendations (16), screening by capillary tests is advisable at the primary level of care, particularly in settings where access to laboratories presents an obstacle. Our findings revealed that glucometers and test strips were available in the majority of primary health care settings and that most women traveled<30 min to their health center. Therefore, screening using capillary tests at the first level would be feasible, more accessible, and could contribute to reducing delays and limiting adverse pregnancy outcomes through timely detection of GDM.

Screening with a fasting blood glucose was generally recommended at the first ANC visit, in accordance with national best practice recommendations for Morocco that suggest a fasting blood sugar in the first trimester (17). The first ANC visit presents an important opportunity to inform pregnant women early on about GDM and raise their awareness about the importance of screening, particularly in the second trimester. However, the information women receive about GDM is often limited, a problem that has also been described in other publications (18, 19).

Adequate information that enables a patient to take her own decisions can positively impact on compliance with screening and treatment (20). This is of particular importance, given that gestational diabetes is neither visible nor painful. A good client–provider relationship is critical for compliance in addition to health system related factors such as accessibility of services that can strengthen adherence and facilitate follow-up (21). Ease of access to providers at the first level of care and their knowledge of patients’ individual and family context are advantages of providing care through primary health care facilities (22). In Morocco, general practitioners of health centers are already in charge of patients with chronic diabetes, but their role in GDM is generally restricted to referring pregnant women with this condition to a specialist. This divide between non-communicable diseases and maternal health presents a substantial barrier to the management of GDM (23).

Endocrinologists are often located in urban areas and have to deal with an already high patient load. Therefore, decentralization of screening and at least initial management of uncomplicated cases through the primary health care level could limit specialist involvement to complicated patients. This would reduce waiting times and delays for affected women, a problem also known in high income settings (18), as well as possible dropouts and loss of patients to follow-up after being referred (23).

Our study is the first of its kind to present the situation of GDM screening and management at district level in Morocco. However, its quantitative findings are limited because they rely on what has been documented by health care providers in ANC cards and patient files. Therefore, practices that were performed, but not documented are not captured in these data. However, we tried to circumvent this limitation by triangulating information with qualitative findings.

## Conclusions

Screening for gestational diabetes and management of uncomplicated cases through the primary health care level could ease access to care and facilitate the surveillance of affected women within the continuum of care. Strengthening the role of the primary level of care in GDM management could contribute to the prevention of future diabetes in affected mothers and their children and result in a posteriori diagnosis of GDM becoming history in Morocco.

## Paper context

The lack of knowledge on GDM screening practices was highlighted in a stakeholder meeting in October 2014 and led to the above-mentioned research being conducted. Several delays in screening and in the subsequent management of GDM have been identified and call for further action particularly as hyperglycemia seem to be widespread among ANC attendees in Morocco. Detection and management of uncomplicated forms of GDM through primary health facilities could guarantee a better continuity of care.
